# Identification by Virtual Screening and *In Vitro* Testing of Human DOPA Decarboxylase Inhibitors

**DOI:** 10.1371/journal.pone.0031610

**Published:** 2012-02-23

**Authors:** Frederick Daidone, Riccardo Montioli, Alessandro Paiardini, Barbara Cellini, Antonio Macchiarulo, Giorgio Giardina, Francesco Bossa, Carla Borri Voltattorni

**Affiliations:** 1 Department of Biochemical Sciences “A. Rossi Fanelli”, University of Rome “La Sapienza”, Rome, Italy; 2 Department of Life Sciences and Reproduction, University of Verona, Verona, Italy; 3 Department of Chemistry and Drug Technology, University of Perugia, Perugia, Italy; Aston University, United Kingdom

## Abstract

Dopa decarboxylase (DDC), a pyridoxal 5′-phosphate (PLP) enzyme responsible for the biosynthesis of dopamine and serotonin, is involved in Parkinson's disease (PD). PD is a neurodegenerative disease mainly due to a progressive loss of dopamine-producing cells in the midbrain. Co-administration of L-Dopa with peripheral DDC inhibitors (carbidopa or benserazide) is the most effective symptomatic treatment for PD. Although carbidopa and trihydroxybenzylhydrazine (the *in vivo* hydrolysis product of benserazide) are both powerful irreversible DDC inhibitors, they are not selective because they irreversibly bind to free PLP and PLP-enzymes, thus inducing diverse side effects. Therefore, the main goals of this study were (a) to use virtual screening to identify potential human DDC inhibitors and (b) to evaluate the reliability of our virtual-screening (VS) protocol by experimentally testing the “in vitro” activity of selected molecules. Starting from the crystal structure of the DDC-carbidopa complex, a new VS protocol, integrating pharmacophore searches and molecular docking, was developed. Analysis of 15 selected compounds, obtained by filtering the public ZINC database, yielded two molecules that bind to the active site of human DDC and behave as competitive inhibitors with K_i_ values ≥10 µM. By performing *in silico* similarity search on the latter compounds followed by a substructure search using the core of the most active compound we identified several competitive inhibitors of human DDC with K_i_ values in the low micromolar range, unable to bind free PLP, and predicted to not cross the blood-brain barrier. The most potent inhibitor with a K_i_ value of 500 nM represents a new lead compound, targeting human DDC, that may be the basis for lead optimization in the development of new DDC inhibitors. To our knowledge, a similar approach has not been reported yet in the field of DDC inhibitors discovery.

## Introduction

Parkinson's disease (PD) is the most extensively studied pathology within a group of syndromes called “motor system disorders”, whose etiology can be traced back to the loss of dopaminergic neurons of the *substantia nigra* in the midbrain [1]. Main symptoms of PD include tremors, rigidity, bradykinesia and postural instability; other frequently observed symptoms include depression and other psychiatric disorders, difficulty in swallowing, chewing, and speaking. Early symptoms of PD are usually subtle and occur gradually after 50 years of age. As the symptoms become more severe, patients progressively encounter difficulties in walking, talking, or even completing the simplest tasks; usually, this condition interferes strongly with most daily activities.

At present there is no cure for PD, but a variety of palliatives reducing the severity of disease symptoms exists [2]. In order to replenish dopamine levels at the central nervous system (CNS), L-Dopa is usually administered. The latter is converted to dopamine by Dopa decarboxylase (DDC, E.C. 4.1.1.28), a pyridoxal-5′-phosphate (PLP)-dependent enzyme, which is abundant in the CNS and in the kidney [3]. DDC from pig kidney has been widely characterized with respect to reaction and substrate specificity [4], [5], spectroscopic features of the internal aldimine and of enzyme-intermediate complexes [6], [7], [8], and the role played by residues at or near the active site in the catalysis [9], [10], [11], [12]. Moreover, the crystal structures of DDC, both ligand-free and in complex with the antiParkinson drug carbidopa, have been solved [13].

Although administration of exogenous L-Dopa to PD patients compensates, at least transitorily, for deficiency of dopamine synthesis and often provides dramatic relief from the main symptoms, only 1–5% of L-Dopa reaches the dopaminergic neurons of the brain, being the major part metabolized by the peripheral DDC. Therefore, in order to increase the amount of L-Dopa in the CNS, DDC inhibitors unable to cross the blood-brain barrier (BBB) are usually co-administered with L-Dopa. In this way, not only greater amounts of L-Dopa can reach the brain, thereby substantially increasing its level, but also side effects, either dopamine-related or due to a high concentration of L-Dopa in the blood stream, are diminished [1]. The most commonly used DDC inhibitors in the treatment of PD are carbidopa (L-α-methyl-α-hydrazino-3,4-dihydroxyphenylpropionic acid, MK 485) and benserazide (trihydroxybenzylhydrazine seryl derivative, Ro-4-4602). Pharmacokinetic and metabolic studies in animals and humans have shown that benserazide is completely metabolized before it reaches the arterial blood and that the main metabolic pathway consists of the scission of the molecule between serine and trihydroxybenzylhydrazine (Ro 4-5127) [14]. Thus, it is likely that trihydroxybenzylhydrazine represents the actual DDC inhibitor. Indeed, while benserazide is not a powerful DDC inhibitor [15], carbidopa and trihydroxybenzylhydrazine, both substrate analogs endowed with a substituted hydrazine function, have been found to bind to pig kidney DDC by forming a hydrazone linkage with PLP and work as powerful irreversible DDC inhibitors [16], [17]. Nevertheless, because hydrazine derivatives can react with free PLP and PLP-enzymes, these inhibitors are not entirely selective for DDC, thus resulting in adverse side effects [18], [19]. Although the crystal structure of DDC has been solved ten years ago, no structure-based design studies have been reported to date. Thus, in order to identify competitive and highly selective DDC inhibitors, we decided to undertake a virtual screening (VS) approach combined with *in vitro* binding experiments.

As a starting point, the structure of pig kidney DDC (∼90% sequence identity with human DDC) in complex with the inhibitor carbidopa (PDB code: 1JS3 [13]) was used to identify the essential features required for DDC binding. Then, a pharmacophore model was generated and validated using an in-house built database of known active and inactive DDC inhibitors, derived from Hartman et al. [20]. The pharmacophore model was first used to filter the lead-like and the drug-like subsets of the public ZINC database (∼8 million compounds [21]) which are tailored to an extended Lipinski's rule of five [22]. Compounds satisfying the pharmacophoric requirements were then instrumental to run docking studies. Hence, compounds showing the highest binding scores were selected, and tested *in vitro* for their ability to bind and inhibit purified recombinant human DDC. The compounds with the highest inhibitory activity were used to perform a second similarity-based filtering of the public ZINC database to retrieve analogs in order to expand the new classes of DDC inhibitors. The *in vitro* testing revealed that 9 hits sorted out from the second screening inhibit human DDC in a competitive mode with K_i_ values in the range 2–15 µM. Subsequently, from a substructure search using the core of the most active compound, a molecule with a K_i_ value of 500 nM emerged as a promising candidate for further lead optimization.

## Results

### Virtual Screening

On the basis of the crystal structure of pig kidney DDC (PDB code: 1JS3) in complex with the anti-Parkinson drug carbidopa, the key chemical features responsible for the critical binding interactions were initially derived. A pharmacophore query was used to build a 8-points pharmacophore hypothesis (PH) containing: (1) an aromatic centroid (F1, Aro) located at the geometric center of the catechol ring and its normal projection (F2, PiN), which points at Lys303, (2) a hydrogen bond donor feature (F3, Don) located on the *para*-hydroxyl substituent of the catechol moiety and its projection (F4, Don2), which points at the side chain of Thr82, (3) a hydrogen bond donor feature (F5, Don) on the *meta*-hydroxyl substituent of the catechol moiety and its projection (F6, Don2), which points at the phosphate moiety of the PLP cofactor, and (4) a hydrogen bond acceptor feature (F7, Acc) located on the oxygen of the carboxylate group and its projection (F8, Acc2), which points at the side chain of His192. Finally, in order to take into account the shape of the active site of DDC, a steric constraint (excluded volume) was derived from the DDC-carbidopa complex and included in the PH. Such features are summarized in Table 1.

**Table 1:** Chemical features in the PHs.

Atoms	Feature	pig DDC - carbidopa	Feature	Final PH
Catechol ring	F1	Aro (1)^E^	C1	Aro (1.57)^E^
	F2	PiN (1.4)	C2	PiN (1.58)
*Meta*-OH	F3	Don (1)	C3	Don (1.28)^E^
	F4	Don2 (1.4)	C4	Don2 (1.49)
*Ortho*-OH	F5	Don (1)	C5	Don (1)
	F6	Don2 (1.4)	C6	Don2 (1.2)
Carboxylate	F7	Acc (1)	C7	Acc (1.49)
	F8	Acc2 (1.4)	C8	Acc2 (1.37)
Active compounds			C9	Acc2 (1.58)^E^
			C10	Acc2 (1.29)

The table summarizes the type of chemical feature, its location in the initial PH, derived from the pig kidney DDC – carbidopa complex, and in the final PH. The features which were regarded as essential are shown. Numbers in parenthesis indicate the sphere radii of each feature (Å).

E Essential.

**Figure 1:**
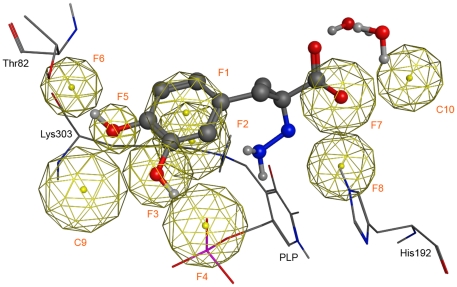
Pharmacophore hypothesis of a competive inhibitor of DDC, superimposed on the 1JS6 – carbidopa complex. The chemical features of the final pharmacophore essential for the binding of a competitive inhibitor to DDC are shown as spheres. Carbidopa is represented as balls and sticks. PLP, water molecules and residues interacting with carbidopa are also shown and labeled.

Then, within a set of already experimentally tested compounds [20], those showing no chirality center were included in a training set. On the basis of assays performed on a pig kidney extract [20], they were clustered in inactive (31 compounds causing a loss of DDC activity <10% at 2.2 mM concentration), poorly active (33 compounds causing a loss of DDC activity ≥10% at 2.2 mM concentration), moderately active (29 compounds causing a loss of DDC activity ≥10% at a concentration in the range 220–440 µM), and highly active (9 compounds causing a loss of DDC activity ≥10% at a concentration <110 µM). All active molecules (i.e., poorly, moderately and highly active compounds) were analyzed with the previously developed PH and the conformations of the matching molecules were processed in order to retrieve consensus chemical features (coordinates, radius and feature type). The latter, in turn, were included in the PH (as detailed in the “Materials and Methods section”). For this purpose, the annotation points of the active compounds matching the PH were clustered in ten chemical features (C1–C10, Table 1). C1–C8 coincided with F1–F8 in terms of position (root mean square deviation (RMSD): 0.13 Å) and type of feature, even if the radii of the spheres were slightly different (Table 1). Moreover, two new chemical features were identified: a hydrogen bond acceptor projection feature (C9) roughly pointing at His302 and a hydrogen bond acceptor projection feature (C10) pointing at two structural water molecules (hydrogen bonded to the carboxylate moiety of carbidopa in the DDC-carbidopa complex). The two new projection features, C9 and C10, were then added to the initial PH, and the radii of all the chemical features, F1 to F8, were subsequently adapted according to the values calculated by the consensus function for the C1–C8 features. Therefore, in order to assess which features could be regarded as essential for the subsequent pharmacophore searches (PSs), we generated several alternative PHs, each containing all but one feature, and compared their performance by carrying out PSs of the final training set (initial training set plus 1950 decoys; see “Materials and Methods section” for further details). The final obtained PH (Fig. 1), in which F1, F3 and C9 were set as essential, retrieved 48% active (30% poorly active, 55% moderately active and 88% highly active) and 6% inactive compounds, thus clearly showing a marked improvement as compared to the initial PH, with 27% active (12% poorly actives, 34% moderately active and 55% highly active) and 3% inactive compounds.

**Figure 2:**
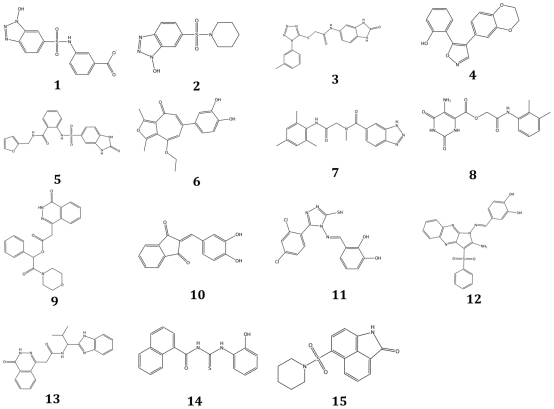
Chemical structures of selected compounds from the first VS screening.

The final PH was then used to filter the drug-like and lead-like subsets of the ZINC database [21]. This PS retrieved ∼280000 drug-like and ∼180000 lead-like molecules, matching at least 6 chemical features. These virtual hits underwent a second docking-based filtering by means of the Dovis docking tool [23], [24]. All the potential hits (∼460000) were docked into the active site of DDC, and the normal distribution of their scores (reported as predicted pK_i_ values) was derived. The distributions of scores for lead-like and drug-like molecules showed a mean pK_i_ of 5.30±0.62 and 5.57±0.81, respectively (Fig. S1 and S2). 2391 drug-like and 2994 lead-like molecules with significant scores (≥2 standard deviations (SDs from mean) were selected and resubmitted to a further docking step by means of a second docking tool, Autodock Vina [25]. 1496 and 1763 drug-like and lead-like compounds, respectively, showed poses comparable when docked with both Vina and Dovis (RMSD value ≤2 Å). Of these, 17 lead-like and 44 drug-like molecules (whose ZINC code and final ranking are reported in Supplementary Tables S1 and S2) fulfilled also the restraints imposed by the final PH. Then, the similar compounds were clustered, and, on the basis of the predicted pK_i_ and the commercial availability, the most potent of each group was chosen. This protocol gave us 7 drug-like and 8 lead-like molecules that were subjected to *in vitro* testing. For each compound the chemical structure is reported in Fig. 2 and the number of matched features of the final PH in Table 2.

**Table 2:** Lead-like (compounds 1, 2, 4, 6, 8, 10, 14, 15) and drug-like (compounds 3, 5, 7, 9, 11, 12, 13) molecules selected by the VS.

	ZINC code	Ambinter	MW(Da)	Matched features	K_i_^a^K_i′_^b^(µM)	BE	Inhibition profile
**1**	02654612	T5246255	333	5	106±7^a^	11.9	Competitive
**2**	03324540	T0516-9932	282	5	390±30^a^	12.1	Competitive
**3**	05936951	T5347527	381	4	n.d.	-	n.d.
**4**	00408890	STOCK1N-01831	295	4	n.d.	-	n.d.
**5**	05252718	T5607516	412	4	59.8±5.8^a^	10.2	Competitive
**6**	00111962	STOCK1N-14396	326	7	n.d.	-	n.d.
**7**	12603254	T5999596	351	6	n.d.	-	n.d.
**8**	03243721	T0511-4207	332	5	n.d.	-	n.d.
**9**	05185571	T6014881	407	5	n.d.	-	n.d.
**10**	00492694	STOCK1S-02871	266	7	6.03±0.55^a^23.1±1.1^b^	-	Mixed
**11**	08042801	PB-05725216	381	6	10.7±2.4^a^	13.0	Competitive
**12**	01874906	STOCK3S-99912	459	9	19.4±1.4^b^	-	Noncompetitive
**13**	07986167	T5522252	375	5	n.d.	-	n.d.
**14**	00342069	STK059041	322	5	n.d.	-	n.d.
**15**	00134865	F0433-0503	316	5	n.d.	-	n.d.

The table summarizes, for each compound, the ZINC-code, the Ambinter-code, the molecular weight, the number of matched features of the final PH, the experimental K_i_ and/or K_i′_ values, the BE, and the inhibition profile. n.d., not determined.

### Inhibition and binding analysis

The 15 compounds resulting from the virtual screening were tested for their ability to bind to and inhibit human recombinant DDC. At a concentration in which their solubility was maximum, compounds **6**, **8**, **13**, and **15** do not inhibit DDC activity, compounds **3**, **4**, **7**, **9** and **14** exhibit weak DDC inhibition activity (8–25%), while the compound **12** exerts an inhibitory effect of DDC activity of ∼55%. On the other hand, the remaining compounds **1**, **2**, **5**, **10** and **11** inhibit DDC by 70% or more. An IC_50_ analysis performed on the latter molecules identified five inhibitors of human DDC with well-behaved IC_50_ curves giving IC_50_ values equal to 570±66 µM, 1500±220 µM, 124±97 µM, 12.8±0.4 µM, and 27.3±1.6 µM for compounds **1**, **2**, **5**, **10** and **11**, respectively. The IC_50_ curve of compound **11** is shown in Fig. S3A. These molecules and also compound **12** were further analyzed in order to identify the type of inhibition. Compounds **1**, **2**, **5**, **11** behave as typical reversible inhibitors with competitive inhibition, compound **10** with mixed inhibition, and compound **12** with noncompetitive inhibition. The inhibition constant values (K_i_ and/or K_i′_) for each of these inhibitors are reported in Table 2. None of the competitive inhibitors changed the absorbance spectrum of free PLP. On the other hand, the addition of any of the competitive inhibitors to human DDC induced a modification of the CD spectrum which, like in the pig kidney enzyme [26], is characterized by positive dichroic bands at 420 and 335 nm. The modification consists of a decrease of the 420 nm positive signal and a concomitant increase of the 335 nm positive band. The fractional changes in the magnitude of the 420 nm or the 335 nm dichroic band as a function of the compound **1**, **2**, **5** and **11** concentrations were used to calculate the K_D_ for the inhibitor-DDC complexes formation. The K_D_ values were found to be 240±68 µM, 516±126 µM, 54.9±9.8 µM and 16.1±2.1 µM, for compounds **1**, **2**, **5**, and **11**, respectively, in good agreement with the corresponding K_i_ values. Taken together, these data strongly suggest that all the competitive inhibitors bind to the human DDC active site.

**Figure 3:**
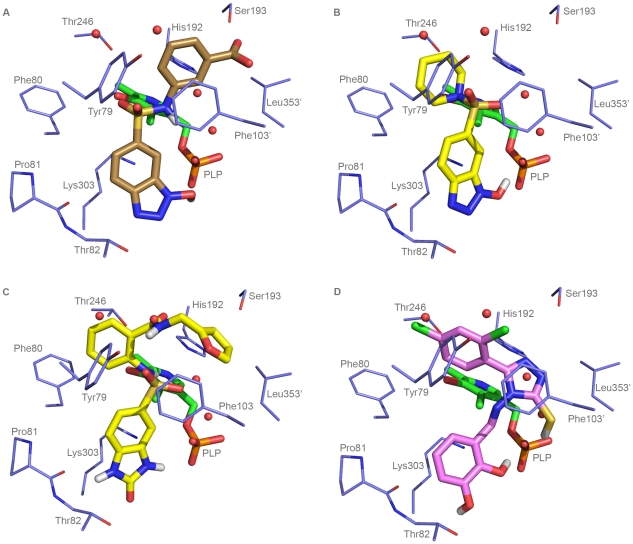
Predicted binding mode of compounds selected after the first VS. The figure shows the binding mode of (A) compound **1**, (B) compound **2**, (C) compound **5**, and (D) compound **11**. Residues and water molecules interacting with the inhibitors are also shown. Figure was rendered with Pymol (http://www.pymol.org/).

As shown in Fig. 3, the computational docking for compounds **1**, **2**, **5**, and **11** gives the following clues regarding the possible binding mode:

#### Compound 1 (Fig. 3A)

The 1-hydroxybenzotriazole ring of this compound shows a cation - π interaction with Lys303. The nitrogen atom and the hydroxylic group of the 1-hydroxybenzotriazole ring are hydrogen bonded to Thr82 and to the phosphate of the PLP cofactor, respectively. The sulfonamidic nitrogen atom interacts with one of the water molecules bridging the phosphate group of PLP and His192. Moreover, the 3-carboxy-phenyl ring displaces the second water molecule, and interacts with Phe103′ (showing a parallel-displaced stacking arrangement) and Leu353′ (accent indicates residues of the active site provided by the adjacent monomer). Finally, the carboxylic group of this ring is hydrogen bonded to Ser193.

#### Compound 2 (Fig. 3B)

The 1-hydroxybenzotriazole ring interacts similarly to the one of compound **1**. One of the oxygen atoms of the sulfonamidic group is hydrogen bonded with the two water molecules interacting with the carboxylic group of carbidopa in the carbidopa-DDC-complex. The piperidinic ring interacts hydrophobically with the residues Tyr79, Phe80 and Thr246.

#### Compound 5 (Fig. 3C)

The 2-carbonyl-2,3-dihydro-benzimidazole ring of this compound shows a cation- π interaction with Lys303, and its nitrogen and carbonyl atoms interact with the amide carbonyl of Pro81 and with Thr82, respectively. Furthermore, the oxygen atoms of the sulfonamidic group interact with several water molecules, which interact with the carboxylic group of carbidopa in the carbidopa-DDC complex. In particular, one of the oxygen atoms is hydrogen bonded to the water molecules bridging the phosphate group of PLP and His192, and the other sulfonamidic oxygen atom is hydrogen bonded to one of the two solvent exposed water molecules. The amidic carbonyl moiety interacts with His192 and a water molecule. Finally, the two aromatic rings, benzene and furan, show hydrophobic interactions with Phe103′, Leu353′, Tyr79, Phe80 and Thr246.

#### Compound 11 (Fig. 3D)

The aromatic catechol ring of this compound approximately overlaps with the catechol ring of carbidopa (and, possibly with the one of L-Dopa) and, similarly to the latter, it shows a cation- π interaction with Lys303. The *ortho* and *meta* hydroxyl groups of the catechol ring are hydrogen bonded to the phosphate moiety of the PLP cofactor and to Thr82, respectively. The catechol is connected to a 3-mercapto-1,2,4-triazole heterocycle through a rigid methylidene-amino moiety. The N1 atom of the heterocycle is hydrogen bonded to His192, and the thiol group to the phosphate moiety of the PLP cofactor. The heterocycle is well positioned to displace the water molecules (which were found to interact with carbidopa in the crystal structure of the carbidopa-DDC complex; PDB code: 1JS3) bridging the phosphate group of PLP and His192. The triazole ring and the 2,4-dichloro-phenyl ring show hydrophobic interactions with several residues located at the opposite sides of the active site entrance, namely Phe103′ and Leu353′, as well as Tyr79, Phe80 and Thr246. Moreover, the triazole ring and Phe103′ show a parallel-displaced stacking arrangement.

### Second Screening (Similarity Search)

**Figure 4:**
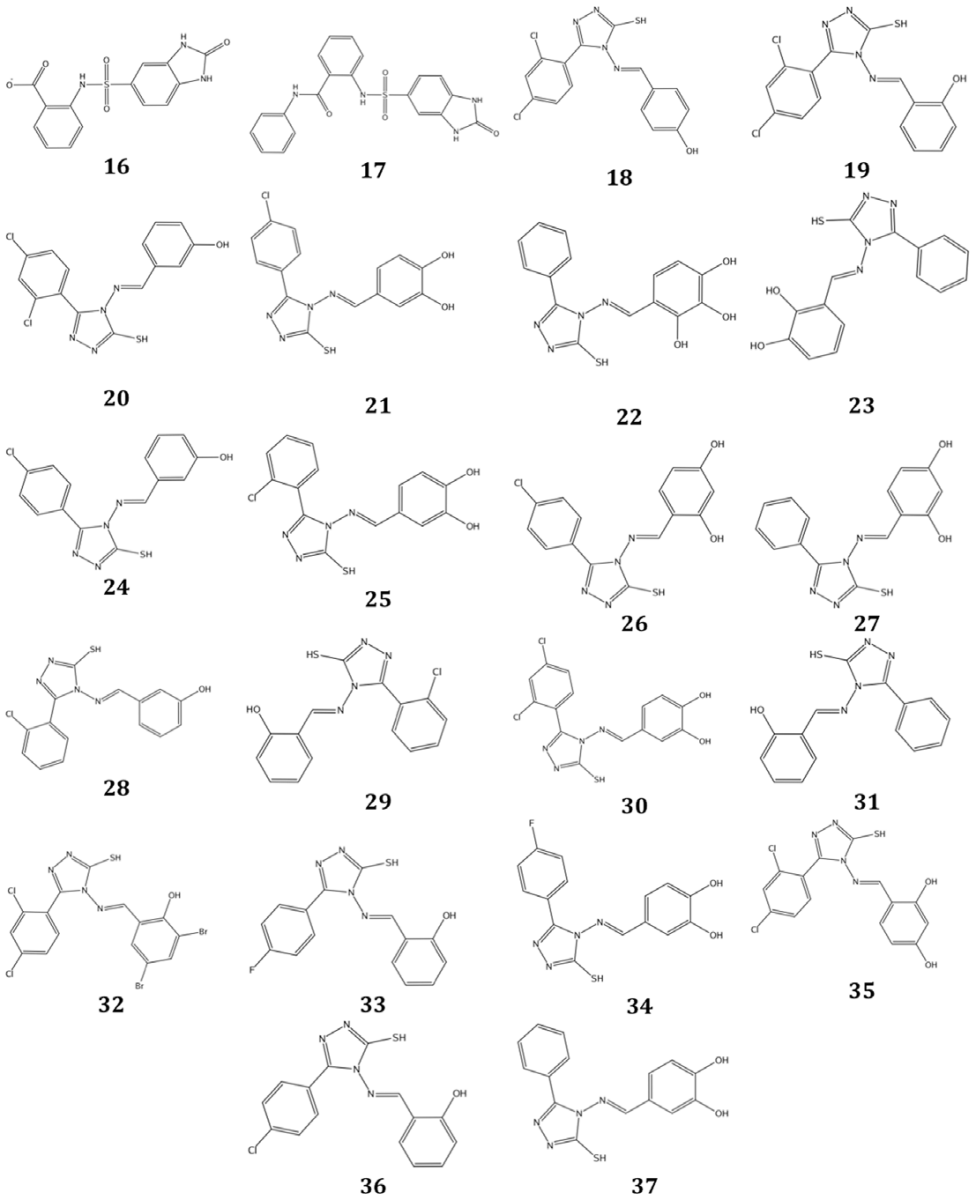
Chemical structures of selected compounds from the secondVS screening.

Following the initial identification of active compounds, a similarity search was carried out by using the most potent hits (compounds **5** and **11**) as query. First, starting from the ZINC database of small molecules, a fingerprinted database was generated, and then the two identified compounds were used as a query to find similar compounds. To this end, the MACCS structural keys [27] and the Tanimoto coefficient (TC) [28] were used as fingerprint system and similarity metric, respectively. Then, each compound was ranked according to the TC, calculated with respect to the query, and those showing a TC>0.80 were selected and further filtered on the basis of the commercial availability. The candidate molecules related to compound **5** showed variations limited to the 2-(((furan-2-yl)-methylamino)-carbonyl) moiety (namely ZINC03886086 and ZINC22621067). On the other hand, a series of compounds showing variations on substitution pattern of either the hydroxyl groups on the catechol ring or the halogen atoms on the second aromatic ring has been selected among the molecules related to compound **11**. This procedure was adopted in an attempt to obtain information on the structure/activity relationship of the above mentioned positions. To this end, two molecules from the Ambinter database, which did not figure in the ZINC database, were also selected. This procedure gave two molecules related to compound **5** and twenty molecules related to compound **11** (see Table S3 and S4 for the ranking of the selected compounds). All of them, whose structures are shown in Fig. 4, were subjected to *in vitro* testing.

### Inhibition and binding analysis

**Figure 5:**
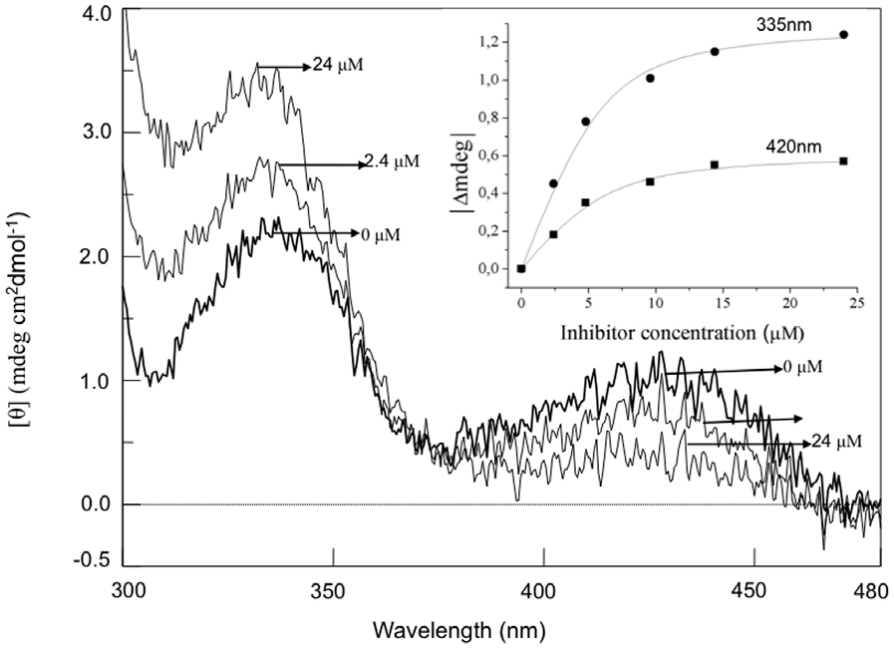
Visible CD spectral changes induced by compound 37 on human DDC. CD spectra of human holoDDC (**—**) and in the presence of the compound **37** at the indicated concentration in 0.1 M potassium phosphate buffer, pH 7.4. Inset: ellipticity change of DDC plus compound **37** at 420 nm and 335 nm as a function of inhibitor concentration. The enzyme concentration was 6 µM.

To probe the reliability of this second screening we tested the effect of the 22 compounds on human DDC activity using the “in vitro” assay. The decarboxylase activity was determined in the absence and in presence of each compound, and IC_50_ values were determined for compounds **17**, **21**, **22**, **23**, **24**, **25**, **28**, **30**, **31**, **34**, **35**, and **37** (Table 3). Dose-response curves of compounds **17**, **21**, **23**, **25**, **34**, and **37** are reported in Fig. S3A. It was not possible to define the IC_50_ values for the remaining compounds since at a concentration in which their solubility was maximum they inhibit DDC activity less than 55%. The analysis of the inhibition profile reveals that compounds **16**, **17**, **18**, **20**, **21**, **23**, **24**, **25**, **28**, **30**, **31**, **34**, and **37** behave as competitive inhibitors of human DDC, while compounds **22**, **27**, **29**, **32**, and **33** inhibit decarboxylase activity in a mixed mode, and compounds **26** and **35** are noncompetitive inhibitors. The K_i_ and/or Ki′ values of these inhibitors are listed in Table 3. Changes in the CD bands of the visible spectrum of DDC induced by the addition of each of the competitive inhibitors are indicative of their binding to the active site of the enzyme. Compounds **21** and **34** cause a decrease of the 420 nm dichroic signal and a concomitant appearance of a positive dichroic signal at 388 nm as well as an increase of the 335 nm dichroic band, while the remaining competitive inhibitors induce a decrease of the 420 nm band and an increase of the 335 nm band. Fig. 5 shows the dichroic spectral changes induced by compound **37** on human DDC. Analysis of the data at 420 nm or at 335 nm as a function of inhibitor concentration yields the following K_D_ values: 17.5±5.1 µM, 28.2±3.2 µM, 2.5±0.7 µM, 4.7±0.5 µM, 2.4±0.1 µM, 21.1±3.5 µM, 25.1±3.5 µM, 31.7±3.4 µM, 7.1±0.9 µM and 1.5±0.2 µM (inset of Fig. 5) for compounds **17**, **20**, **21**, **23**, **25**, **28**, **30**, **31**, **34**, and **37**, respectively. These values are in good agreement with the corresponding K_i_ values. Unfortunately, the K_D_ values for the compounds **16**, **18**, and **24** cannot be measured because of their low solubility. Like the competitive inhibitors of the first series, none of these competitive inhibitors alters the absorbance spectrum of free PLP. In addition, when human DDC complexed with each of the competitive inhibitors was subjected to extensive dialysis against 100 mM potassium phosphate buffer, pH 7.4, the CD spectra of the dialyzed enzymes were identical to that of free DDC. This clearly indicates a reversible binding mode of these molecules.

**Table 3:** Structural analogs of the most potent hit compounds (compounds 5 and 11) obtained after the first screening.

	ZINC-code	Ambinter	MW(Da)	IC_50_(µM)	K_i_^a^K_i′_^b^(µM)	BE	Inhibition profile
**16**	03886086	PB-90199554	335	n.d.	59.8±0.3^a^.	12.7	Competitive
**17**	22621067	PB-251389058	408	16.8±1.7	4.4±1.4^a^	13.1	Competitive
**18**	01183789	PB-90040583	365	n.d.	63.2±3.1^a^	11.5	Competitive
**19**	01183732	PB-90046346	365	n.d.	n.d.	-	n.d.
**20**	01184616	PB-90047186	365	n.d.	36.8±4.2^a^	12.1	Competitive
**21**	00550832	STK103726	347	6.3±0.9	2.3±0.4^a^	16.3	Competitive
**22**	03044871	STK508886	328	6.7±0.9	15.1±0.1^a^46.7±0.5^b^	-	Mixed
**23**	-	PB-05713794	312	16.6±.3	5.7±0.1^a^	16.8	Competitive
**24**	00549544	STOCK3S-13636	331	77.1±2.4	120±14^a^	11.9	Competitive
**25**	00520318	STOCK3S-69837	347	15.2±1.3	3.7±0.2^a^	15.7	Competitive
**26**	00548914	STOCK3S-09410	347	n.d.	65.2±−4.1^b^	12.1	Nonconmpetitive
**27**	00344575	STOCK3S-13173	312	n.d.	24.8±1.2^a^43.1±1.9^b^	-	Mixed
**28**	00554457	STOCK3S-64376	331	17±1	14±1^a^	14.7	Competitive
**29**	00553626	STOCK3S-56595	331	n.d.	44.4±2.3^a^208±3^b^	-	Mixed
**30**	01478099	STOCK3S-62307	381	29.8±4.8	12.2±0.4^a^	12.9	Competitive
**31**	00199179	STOCK3S-19327	296	83±12	33.4±5^a^	15.1	Competitive
**32**	-	STOCK3S-20068	523	n.d.	4.5±0.3^a^17±0.2^b^	-	Mixed
**33**	00532465	STOCK4S-26311	314	n.d.	16.5±0.8^a^325±39^b^	-	Mixed
**34**	00530774	STOCK4S-17596	330	11±0.8	10.4±3.3^a^	15.8	Competitive
**35**	01473651	STOCK2S-87581	381	29±2	127±16^b^	-	Noncompetitive
**36**	04898088	STOCK2S-83947	331	n.d.	n.d.	-	n.d.
**37**	00344577	STOCK2S-84925	312	2.4±0.3	1.8±1.1^a^	18.4	Competitive

MACCS structural keys system (BIT_packed) and the Tanimoto coefficient were used as fingerprint system and similarity metric, respectively. The table summarizes, for each compound, the ZINC-code, the Ambinter-code, the molecular weight, the experimental IC_50_, K_i_ and/or Ki′values, the BE, and the inhibition profile. n.d., not determined.

The binding mode of the compounds resulting from the second screening was also investigated by molecular docking means (applying the procedure described in “Materials and Methods Section”). This analysis provided us with the following information:

#### Inhibitors (16 and 17) related to compound 5

**Figure 6:**
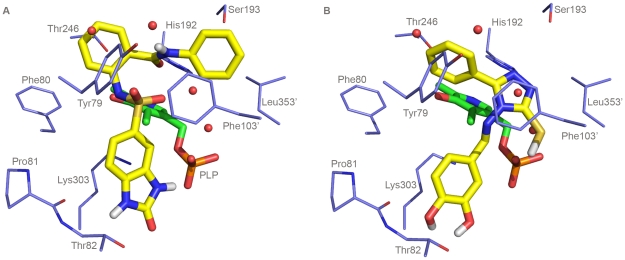
Predicted binding mode of compounds selected after the second VS (similarity search). The figure shows the binding mode of (A) compound **37** and (B) compound **17**. Residues and water molecules interacting with the inhibitors are also shown. Figure was rendered with Pymol (http://www.pymol.org/).

Compounds **16** and **17** (Fig. 6A) are predicted to have binding properties very similar to the ones of compound **5**. This is not unexpected given their structural resemblance. However, it should be noted that (i) the carboxyl group of compound **16** could form a salt bridge with Arg447, (ii) the benzene ring of compound **17**, which replaces the furan ring of compound **5**, is predicted to assume a partial parallel-displaced stacking arrangement with Phe103′, and (iii) the nitrogen atoms of the 2-carbonyl-2,3-dihydro-benzimidazole ring of both compounds, which are slightly translated with respect to compound **5**, are hydrogen bonded to Thr82 and the phosphate group of the PLP cofactor.

#### Inhibitors (18–37) related to compound 11

All these compounds are predicted to have binding features very similar to the ones of compound **11**, even if some differences in the interactions of their hydroxyl groups with the protein and/or the coenzyme can be noted. In Fig. 6B the predicted binding mode of compound 37 is shown.

### Third Screening

**Figure 7:**
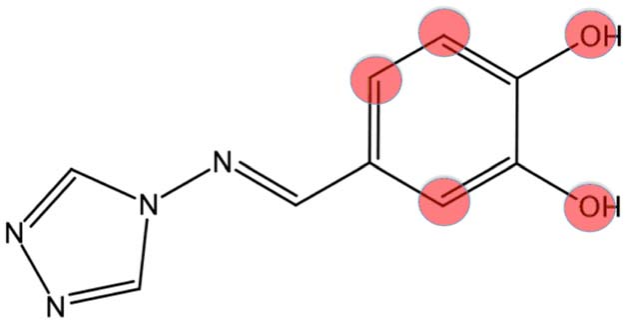
Core of compound 37. The core of compound **37** was used as a query (the Smiles annotation string used is shown in the text), using OpenBabel, to filter the Ambinter database. Positions where no substitutions were allowed during the search are coloured in red.

In order to identify molecules with increased activity and obtain insights into structure-activity relationships, we further expanded the class of active compounds resulting from the second VS by a substructure search carried out using the core of the most active compound **37** (Fig. 7). The OpenBabel substructure search tool (Open Babel v. 2.3.1, 2011) was used to filter the Ambinter database of small molecules, using as query the following SMILES notation (Weininger, 1988): [OH]c2[cH][cH]c(C = Nn1cnnc1)[cH]c2[OH]. Since the most potent compounds obtained in the second VS (see compounds **21**, **37**, **21**) have a catechol ring with *meta*- and *para*- hydroxylic groups, the explicit hydrogens of the unsubstituted benzene carbon atoms and of the hydroxylic oxygen atoms were included in the SMILES notation in order to avoid the selection of compounds with substitution patterns involving these positions.

**Figure 8:**
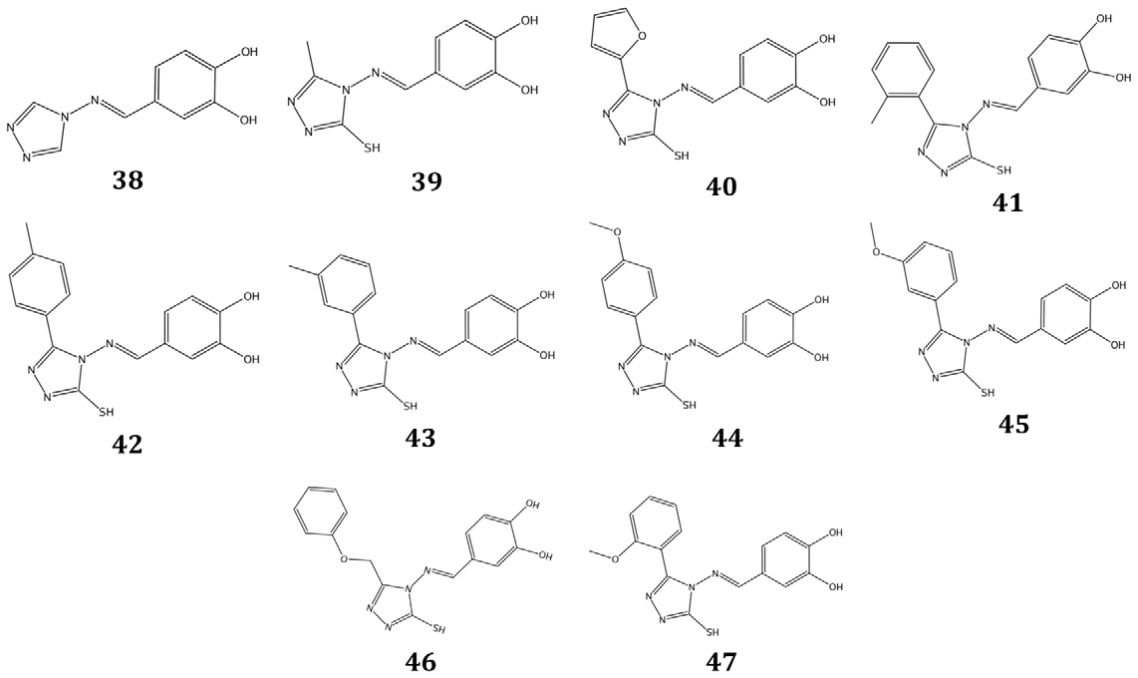
Chemical structures of selected compounds from the thirdVS screening.

The substructure search retrieved 73 hits from the Ambinter database. Among these hits, a series of compounds were selected based on several criteria including i) commercial availability; ii) predicted pose in the active site of DDC, according to docking and modeling analysis; iii) clustering, based on similarity and subsequent removal of redundancy. At the end of this step, 10 compounds, whose chemical structures are reported in Fig. 8, were selected: a compound (**38**) representing the core of compound **37** and a closely related derivative (**39**) showing methyl and thiol substituents at position 3 and 5, respectively, of the 1,2,4-triazole ring; one compound showing a substitution of the benzene ring for a smaller furane ring (compound **40**); six compounds with different substitution patterns of the benzene ring (compounds **41**, **42**, **43**, **44**, **45**, **47**); one compound with a methoxy linker between the 1,2,4-triazole and the benzene ring of compound **37** (compound **46**). All of them were subjected to *in vitro* testing.

### Inhibition and binding analysis

The inhibition activity of these compounds was evaluated by human DDC “in vitro” assay. Compound **47** at 200 µM (the concentration of its maximum solubility) does not inhibit DDC activity. On the other hand, the IC_50_ and the K_i_ values for competitive inhibition of the remaining compounds have been determined and listed in Table 4. Dose-response curves of compounds **39**, **40**, **41**, **42**, **43**, **44**, **45**, and **46** are shown in Fig. S3B. None of these active compounds binds free PLP but, when added to human DDC, they cause CD spectral changes identical to those of compound **37**. The K_D_ values of compounds **38**, **39**, **41**, **42**, **43**, and **45**, determined as above reported, were found to be 24.5±4.4 µM, 9.1±0.3 µM, 6.1±0.5 µM, 2.7±0.5 µM, 2.8±0.5 µM and 1.5±0.3 µM, respectively. These values are very close to the corresponding K_i_. In the case of compounds **40**, **44**, and **46**, the enzyme concentration required for CD measurements far exceeds their K_i_ values, which precludes the determination of the K_D_ values of these molecules.

**Table 4:** Structural analogs of the most potent hit compound (37) obtained after the second screening.

	Ambinter	MW(Da)	IC_50_(µM)	K_i_(µM)	BE
**38**	Amb1808242	204	76.0±5.8	24.5±1.4	22,6
**39**	Amb8754110	250	27.5±3.3	10.8±0.8	19,9
**40**	Amb666646	302	3.5±0.2	1.4±0.1	19,4
**41**	Amb5967895	326	30.1±2.1	9.8±0.5	15,4
**42**	Amb655238	326	5.2±0.4	2.0±0.2	17,5
**43**	Amb685531	326	3.8±0.6	1.9±0.1	17,5
**44**	Amb2470350	342	1.7±0.2	0.5±0.1^a^	18,4
**45**	Amb2472735	342	6.2±0.3	2.0±0.1^a^	16,7
**46**	Amb666103	342	3.5±0.3	1.1±0.1	17,4
**47**	Amb782700	342	>200	n.d.	n.d.

The table summarizes, for each compound, the Ambinter-code, the molecular weight, the experimental IC_50_, K_i_′ values, and the BE. n.d., not determined.

### Ability to cross the Blood-Brain Barrier (BBB) of identified compounds

All identified inhibitors were analyzed to estimate their ability to cross the blood-brain barrier (BBB), according to a simple prediction model developed by Clark *et al.* [29]. According to this model, compounds with predicted values of logBB less than −0.3 are not considered capable of crossing the BBB, while values greater than zero are predictive of concentration in the brain higher than in the blood. All of the identified inhibitors have logBB less than −0.3, and ranging from −0.57 to −1.65, highlighting the poor BBB penetration propensity of these compounds. Moreover, the most active among the compounds related to **5** and **11**, namely compounds **17** and **44**, showed logBB values of −1.24 and −1.30, respectively, comparable with the one of carbidopa that is equal to −1.50.

## Discussion

PD is one of the most common neurodegenerative disorders. It is characterized clinically by parkinsonism (resting tremor, bradykinesia, rigidity, and postural instability) and pathologically by the loss of dopaminergic neurons in the *substantia nigra*. Aromatic hydrazine derivatives (e.g., carbidopa and benserazide) are used in combination with L-Dopa to treat the symptoms of PD or Parkinson-like symptoms. Being unable to cross the BBB, the former drugs work by inhibiting only DDC at the peripheral level. Several side effects (from nausea, hypotension, arrhythmias, and gastrointestinal bleeding to more serious psychiatric symptoms, including auditory and/or visual hallucinations, confusion, psychosis and suicidal thoughts) ensuing from co-administration of L-Dopa with carbidopa or benserazide have been reported [30]. These adverse effects can be ascribed, at least partially, to the mode of action of carbidopa and trihydroxybenzylhidrazine (the *in vivo* hydrolysis product of benserazide) that, by reacting non-enzymatically with free PLP, would cause PLP depletion [18], [19], [31]. It has also been found that the condensation products formed between PLP and hydrazine derivatives are the most potent inhibitors of pyridoxine kinase, an enzyme involved in the biosynthesis of PLP [32], [33]. In addition, it must be pointed out that PD patients treated with L-Dopa and DDC peripheral inhibitors show high levels of plasmatic homocysteine [34], [35] found to be inversely correlated with PLP concentrations in the treated patients [36]. Based on these reports, it is likely that admnistration of L-Dopa in combination with carbidopa or benserazide could be responsible for an alteration of the PLP-dependent metabolism. Thus, there is currently a great interest in the identification of highly potent, reversible and selective DDC inhibitors.

A hierarchical filtering approach was used in this work to identify novel inhibitors of DDC. The hits identified in the initial VS were tested *in vitro* on human DDC. This initial information was used to guide a second round and a third round of VS, in order to retrieve potentially more potent substances and expand the new classes of inhibitors, thereby gaining insights into their structure-activity relationships. In the last years, a lot of effort has been made to improve the rank order in VS approaches. Often, compounds are ranked according to their estimated free energy of binding, and molecular docking is commonly used to this purpose. However, its high computational cost and required time set an upper limit to the amount of compounds that can be processed. One way to solve this issue is to carry out the docking analysis on a subset of the initial database, filtering the latter by other means. Thus, to improve the rank order in VS approaches, pharmacophoric modelling was used in this work, as previously proposed by others [37], [38]. In this way, we were able to select only those compounds (∼10^5^), which show the essential chemical features for DDC binding. During the whole VS process, we didn't make use of additional scoring functions as a consensus [39], [40], [41], other than the ones implemented in Dovis 2.0 and Autodock Vina. However we exploited, in addition to docking score criteria, a pose dependent selection criterium (see “Materials and Methods section”). In this way, final compounds were selected if they 1) showed a comparable docking pose as obtained by two independent search algorithms, 2) were top ranking hits, as assessed by two independent scoring functions, and 3) satysfied the restraints imposed by the pharmacophore model To our knowledge, the VS protocol that we used, in which Pharmacophore-Based Virtual Screening is integrated as a pre-processing and post-processing step with the main docking-based virtual screening step, has never been applied before. Notably, it resulted in a high success rate (5 active compounds out of 15) during the initial *in vitro* testing on human DDC. Such complex VS approach outperformed simpler approaches such as 2D similarity or substructure searching. In fact, when the latter were used with carbidopa as query (by the MACCS structural keys and the TC as fingerprint systems and similarity metric, respectively) on the same drug-like and lead-like subsets, compounds **1**, **2**, **5** and **11** were ranked, according to TC, in positions ≥7.5×10^5^. Therefore, they would have not been identified.

By means of our VS approach we identify three new classes of compounds acting as competitive human DDC inhibitors: (i) compounds **1** and **2**, (ii) compound **5** and its analogs, and (iii) compound **11** and its analogs. These classes show scaffolds composed by two differentially substituted aromatic cycles, connected by a rigid hydrophobic linker with an electron-rich group. The first aromatic ring is represented by a catechol ring (compound **11** and its analogs), or a 2-carbonyl-2,3-dihydro-benzimidazole (compound **5** and its analogs) or a hydroxybenzotriazole (compounds **1** and **2**) bicyclic ring. The linker is represented by a 4-N-[3-mercapto-1,2,4-triazol-4-yl]-carboximidoyl moiety (compound **11** and analogs) or a sulfonamidic group (compounds **1**, **2**, **5** and its analogs). The second ring is represented by a substituted chloro-benzene ring (compound **11** and analogs), or a piperidine ring (compound **2**), a carboxy-benzene ring (compounds **1** and **16**), a 2-(((furan-2-yl)-methylamino)-carbonyl)-benzene ring (compound **5**) or a 2-(((phenyl)-amino)-carbonyl)-benzene ring (compound **17**).

When taking into account the chemical structure as well as the inhibition and binding data of the compounds, simple structure-activity observations can be derived. For molecules related to compound **5**, the substitution of the furan-2-yl-methyl moiety with a benzene ring results into an about 13-fold increase of affinity (compare compounds **5** and **17**), while the substitution of the furan-2-yl-methylamino-carbonyl moiety with a carboxylic group does not improve affinity (compare **5** and **16**). For the molecules related to compound **11**, it can be observed that (i) the shift of the two hydroxyl substituents from the *ortho-meta* to the *meta-para* positions results in compounds with comparable binding affinity (compare compounds **11** and **30**), (ii) the removal of chlorine subtituents from the *ortho-meta*) hydroxy-substituted compound (compound **11**) results in a decrease of K_i_ (compare compounds **11** and **23**), (iii) the removal or substitution of one or two chlorine subtituents from the *meta-para* hydroxy-substituted compound (compound **30**) results in a decrease in K_i_ values in the following order: compounds **30** (*ortho*-*para*-Cl)≈**34** (*para*-F)>**25** (*ortho*-Cl)>**21** (*para*-Cl)≈**37** (unsubstituted), (iv) the removal of a hydroxylic group from the *ortho-meta* (compound **11**) or *meta-para* (compound **30**) hydroxy-substituted compounds results in a increase of K_i_ in the following order: compound **11**≈ compound **30**<compound **20** (*meta*-hydroxy)<compound **18** (*para*-hydroxy)<compound **19** (*ortho*-hydroxy, poorly active), (v) the removal or substitution of chlorine from the mono-*ortho*-hydroxy (compound **19**) and mono-*meta*-hydroxy (compound **20**) compounds results in compounds either of different affinity (compounds **24**, **28**, **31**, **36**) or different inhibition profile (compounds **29**, **32**, **33**). Among those showing a competitive inhibition profile, compound **31** (unsubstituted) and compound **28** (*ortho*-Cl) are the most potent, and (vi) the presence of a hydroxylic-substitution pattern different from that of the catechol moiety, *ortho-meta or meta-para*, or of a mono hydroxylic-substituted ring, results in a deviation from the competitive inhibition profile, i.e., mixed (compounds **22**, **27**, **32**) or noncompetitive (compounds **26** and **35**) inhibition.

According to their predicted binding mode (see results) the three classes of active compounds share some or all of the following key interactions: (i) cation - π interaction with Lys303 and one or two hydrogen bonds with the phosphate of PLP, Thr82 or the carbonyl of Pro81; (ii) hydrogen bond interactions with the active-site water network and/or with His192; (iii) stacking interaction with Phe103′; (iv) hydrophobic interactions with Tyr79, Phe80, Thr246 and Leu353′. The hydrophobic interactions with Tyr79, Phe80, Thr246 and Phe103′ represent two new pharmacophoric features of our developed structure/ligand-based pharmacophore model, and it is noteworthy that the compounds **17**, **21**, **23**, **25**, and **37** showing the lowest K_i_ (<10 µM) fulfill all these binding requirements, while, compound **2**, missing several key interactions, exhibits the highest Ki value (K_i_ = 390 µM). It should be noted that in the spatial structure of ligand free DDC and its complex with carbidopa a flexible loop between residues 328 and and 339 is invisible in the electron density map [13]. Limited proteolysis and site-directed mutagenesis data have indicated that substrate-induced conformational changes occur at this loop and that its closure is an essential step in the decarboxylation mechanism [4], [42]. On the basis of our docked pose it is predicted that this conformationally dynamic loop forms a cleft that accommodates the benzene ring of compound **37** and its derivatives. Although the lack of structural information on this loop does not allow the rational design of this part of the molecule to improve the binding efficiency, the results of out third VS allow to draw some conclusions about the influence of the cleft on inhibitor binding and its ability to accommodate different chemical groups. In particular, we noticed that: (ii) the presence at the 5 position of the thiol substituent, which is predicted to interact via hydrogen-bond with the phosphate tail of PLP, improves binding efficiency as can be appreciated by comparing compound **38** and **39**, (ii) the substitution of the benzene ring for a smaller furane has only minimal influence on activity (compare compounds **37** and **40**), (iii) the analysis of compounds in which the benzene ring is decorated with a methyl (compounds **41**, **42**, **43**) or a methoxy substituent (compounds **44**, **45** and **47**) in *ortho*-, *metha*- and *para*- positions, respectively, allows to infer that, although the cleft is able to accommodate compounds bulkier than **37**, a net preference of *metha*- and *para*- over *ortho*- substitutions could be observed.

In conclusion, this research has allowed the identification of compounds which are predicted do not cross the BBB, and, unlike carbidopa and thrydroxybenzylhydrazine (the *in vitro* hydrolysis product of benserazide) behave as reversible competitive inhibitors with K_i_ values in the low micromolar range. The most potent inhibitor binds human DDC with a K_i_ of 500 nM. Moreover, the finding that they do not interact with free PLP allows us to envisage that these inhibitors could have an intrinsic chemical reactivity lower than that of carbidopa and the metabolic product of benserazide. Thus, the compounds identified and tested here may provide precious guidelines for the drug development process for the PD therapy.

## Materials and Methods

### Materials

L-Dopa, PLP, 2,4,6-trinitrobenzene-1-sulfonic acid, isopropyl-β-D-thiogalactopyranoside, protease inhibitor cocktail (P8849) were Sigma products. DDC inhibitors were purchased from Ambinter (Paris, France). Purity (90% or higher) of these compounds was confirmed on the basis of NMR and/or LCMS data provided by vendors. All other chemicals were of the highest purity available.

### Databases of chemicals

The drug-like and lead-like subsets of the ZINC database [21] were downloaded and used to generate low-energy conformations. The “Conformation import” function of MOE (The Molecular Operating Environment; Chemical Computing Group®, Montreal, Canada) was applied to this purpose. For each compound, the following settings were applied here and thereafter, if not otherwise stated: maximum 250 conformers for each compound; no input filters used; constraints options kept at their default values; only the conformers with strain energy less than 4 kcal/mol retained.

All of the compounds of the training set [20] were built with MOE, energy minimized using the mmff94x force field, and used to build a series of energetically plausible conformations. A set of 1950 decoy molecules, provided by Schrodinger (Schrodinger®, L.L.C., New York), was imported in MOE and series of energetically reasonable conformations were generated for each compound, using the “conformation import” function of MOE. Therefore, the final training set is composed by the union of the initial training set plus decoys.

### Target preparation

Since a potential competitive inhibitor is expected to bind to the internal aldimine of DDC, we made use of PDB structure 1JS6 for pharmacophoric modeling purposes. To this end, 1JS6 was structurally aligned to 1JS3 using the “MOE-ProSuperpose function”; then, we manually inserted carbidopa into the active site of 1JS6, on the basis of the 1JS3 structure. The obtained complex was visually inspected in order to verify the absence of steric clashes between carbidopa and the residues at the active site.

### Pharmacophore Model generation

Pharmacophore modeling calculations were performed using MOE. The MOE PQ function was used in order to build the PH starting from the 1JS6 – carbidopa complex. The “Unified Annotation Scheme” was used to assign the pharmacophoric annotation points (such as H-bond donor, H-bond acceptor, hydrophobic). Chemical features (coordinates, type of chemical feature and sphere radius) corresponding to atoms or groups of atoms of carbidopa were inserted in the PH. MOE's default values for the radii of atom-based chemical features (1 Å) and projections (1.4 Å) were used. An excluded volume, comprising residues at the active site of DDC located within a radius of 15 Å from any atom of carbidopa, was inserted in the PH.

The active compounds of the training set were analyzed by PS using the PH. During each PS the “partial match” option was activated and the number of chemical features to be matched was always set to “at least three” (default setting, if not otherwise stated). The aromatic centroid feature of the catechol ring of carbidopa was set as “essential”. For each compound, all of the matching conformations were selected. For each of the selected conformation, the number of matched features and the RMSD between the centers and the corresponding annotation points were derived. The conformations for each selected compound were sorted by decreasing number of matched features and increasing RMSD; for each compound only the highest ranked conformation was selected. This procedure was applied also in the following PSs.

Of the 71 active compounds analyzed, all satisfied the restraints imposed by the PH, and they were subsequently subjected to the “pharmacophore consensus function” of MOE. The “pharmacophore consensus function” suggests, from a set of structurally aligned molecules, common chemical features (coordinates, radius and feature type) that can be in turn inserted in a PH. When the “pharmacophore consensus function” was used, the following settings were applied: tolerance radius set to 1, the consensus score set to “weighted conformations” and the threshold set to 30%. Thus, the PH was modified according to the new data obtained from the pharmacophore consensus analysis: new identified features were added to the PH and the radii of the spheres of all the chemical features were increased/decreased accordingly.

In order to assess which feature of the PH should be regarded as essential for the subsequent PSs, we generated several alternative PHs, by discharging each time one feature. Then, we evaluated each PH for its ability to rank active and inactive compounds. To this end, we first derived a new dataset of compounds, merging the training set and the decoys. Then, for each PH, we measured the fraction of active and inactive compounds that were present amongst the best 3.46% ranking hits (corresponding to the ideal situation, in which all 71 active compounds are ranked in the first 71 positions of the ranked hits), according to the PS. When the PH with all the chemical features was used, 48% of the active molecules and 10% of the inactive molecules of the training set were retrieved in 3.46% of the top ranking hits. Among the generated alternative PHs, the one showing the highest decrease in terms of retrieved active compounds lacked the C9 feature. Moreover, the PHs showing the highest increase in terms of retrieved inactive compounds were those lacking F3 (16%) and C10 (16%).

Finally, to verify the effects of the changes brought to the PH in the previous steps, we evaluated the ability of the PH to rank active and inactive compounds when used with its initial settings (derived from the 1JS6 – carbiDOPA complex) and with the final settings (see Table 1). To this end, the same database was used as before and we measured the fraction of active (poorly active, moderately active and highly active) and inactive compounds, which were present amongst the best 3.46% ranking hits, according to the PS.

### Docking settings

The drug-like and lead-like compounds retrieved from the PS were docked into the active site of DDC. To this end Dovis 2.0 [23], [24] was applied. Initially, the complex PLP-carbidopa was re-docked in the active site of DDC (PDB code: 1JS3), in order to optimize the parameters of the docking program to be used during the high-throughput docking. To this end, 1JS6 was structurally aligned to 1JS3 using the “MOE-ProSuperpose function”; then, we manually inserted carbidopa into the active site of 1JS6, on the basis of the 1JS3 structure. The obtained complex was visually inspected in order to verify the absence of steric clashes between carbidopa and the residues at the active site. To this end, an energy grid with 40×40×40 (numbers refer to the number of grid points in xyz), centered on the ε-amino group of the active site Lys303 of pig kidney DDC, and a 0.375-point spacing was used. Atom-specific affinity maps were then computed for each atom observed in the ligand, and a distance-dependent dielectric constant was adopted. Several docking simulations were initially run. During each simulation, grid dimension and center values were systematically varied, while other parameters were maintained at their default values, in order to find which combination of values was able to reproduce the binding pose of carbidopa bound to PLP present in the three dimensional structure. The selected values for grid dimensions and center were respectively 36×36×36 and *x* = 43.573 *y* = 37.127 *z* = 68.616 (coordinates are referred to the 1JS3). During the high-throughput docking, the Lamarckian Genetic Algorithm was chosen as search algorithm, using the following values: number of individuals in population, 150; maximum number of energy evaluation, 250000; maximum number of generation, 27×10^3^; rate of gene mutation, 0.02. Water molecules were excluded from the docking calculations. All other parameters were kept at their default values.

After initial parameter optimization, the active compounds of the training set plus decoys were docked in the active site of DDC by means of Dovis 2.0, and ranked according to their predicted energy of binding. Only 11% of highly active compounds were retrieved within approximately the first 15% of the screened database. Then, in an attempt to improve the docking step, we applied a procedure involving two independent docking tools (Dovis 2.0 and AutoDock Vina), and we considered only those compounds showing a similar pose (RMSD<2 Å). Moreover, compounds were ranked according to the mean of their predicted energy of binding. By applying this second approach, 44% of highly active and only 3% of inactive compounds were retrieved within approximately the top-ranking 15% of the screened dataset. Therefore, the latter approach was adopted for the complete VS protocol.

When Autodock Vina [25] was used, the same grid parameters described previously were adapted, the exhaustiveness option was set to 8, and all other options were kept at their default values. Since a potential competitive inhibitor is expected to bind to the internal aldimine of DDC, we made use of PDB structure 1JS6 for subsequent docking purposes.

### Post-filtering phase

A post-filtering step, based on the initial PH hypothesis, was coupled to the main docking-step. The latter, introducing additional structural knowledge on the “hot spots” of the active site, increased the performance of the VS protocol. Indeed, when the training set plus decoys was analyzed by the complete VS-protocol (docking plus pharmacophore search), 44% of highly active, 10% of moderately active, 3% of poorly active and 6% of inactive compounds were ranked in the top 0.6% of the screened database.

During the post-filtering phase, the poses of the docked drug-like molecules obtained in the previous docking-based virtual screening step were ranked according to their score, obtained with the scoring function [24] implemented in Dovis 2.0. The mean and standard deviation of the scores were then calculated, and those compounds scoring at >2 standard deviation from mean were selected and re-docked with Autodock Vina. Only those compounds showing a similar (RMSD<2 Å) docked pose, as assessed by Dovis 2.0 and AutoDock Vina, were kept. Finally, the selected poses were filtered with the previously developed PH with the “absolute position” option activated. In this way, when analyzed during PS, the poses were not allowed to be translated or rotated, and only those in agreement with the PH were kept. Lead-like molecules were processed similarly.

### Assessment of the binding efficiency (BE) and BBB of selected compounds

When assessing the druggability of newly identified molecules, it is useful to monitor their molecular weight. This parameter relates to a contrasting trend: (1) an important increase in molecular weight is often required to reach the appropriate level of potency, (2) key properties that determine the druggability of a molecule tend to worsen when the molecular weight increases over a certain point [43]. Therefore, when establishing the viability of a molecule both potency and molecular weight need to be monitored and well balanced. The concept of BE represents a suitable means of assessing this relationship; it is, for a given compound, a measure of the binding energy per unit of mass relative to its target. Several definitions have been proposed, but throughout this work the following has been be used:
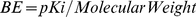
(1)being K_i_ expressed in molarity (micromoles/L), and the molecular weight in kDa. It has been observed that 90% of lead molecules and drugs have efficiencies above 12.4 and 14.7, respectively [43].

All identified inhibitors were analyzed to estimate their ability to pass the BBB according to a simple prediction model developed by Clark [29], which takes into account the octanol/water partition coefficient (logP), calculated according to Moriguchi (MlogP; [44]), and the topological polar surface area. Moreover, values for MlogP topological polar surface area were calculated using the AlogPS online software [45] and MOE, respectively. Predicted log BB was calculated using the formula:
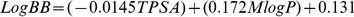
(2)Compounds with predicted values of logBB less than −0.3 are not considered capable of crossing the BBB, while values greater than zero are predictive of concentration in the brain higher than in the blood.

### Expression and Purification of Human DDC

Human DDC was expressed and purified by mean of the procedure previously described [46]. The enzyme concentration was determined by using an ε_M_ of 1.3×10^5^ M^−1^ cm^−1^ at 280 nm [26]. PLP content of wild-type was determined by releasing the coenzyme in 0.1 M NaOH and by using ε_M_ = 6600 M^−1^ cm^−1^ at 388 nm.

### Assay of the inhibitory activity

DDC activity was determined by measuring the production of dopamine with a spectrophotometric assay outlined by Sherald *et al.* [47] and modified by Charteris and John [48]. The reaction was carried out in 0.1 M potassium phosphate buffer pH 7.4 at 25°C for 1 min and stopped by inactivation of the enzyme at 100°C for 90 sec. The inhibitory assay was performed by measuring the decarboxylase activity of DDC with 50 nM enzyme, 0.1 mM L-Dopa, and 10 µM PLP in the presence and in the absence of a fixed amount of each compound. Various amounts of dimethyl sulphoxyde (DMSO) were necessary to solubilize the compounds in 100 mM potassium phosphate buffer, pH 7.4. In any case, the final DMSO concentration in the assay mixture was kept lower than 1% (v/v), i.e., under experimental conditions in which decarboxylase activity is not affected. Dose-response curves were generated to determine the concentration required to inhibit 50% of decarboxylase activity (IC_50_). Compounds were evaluated at 9 concentrations, and each in duplicate. IC_50_ values were derived by nolinear regression analysis. The related inhibition type of the compounds on human DDC was identified from Lineweaver–Burk plots, and K_i_ and/or K_i′_ values for the inhibitors tested were calculated by the software Origin 7.0.

### Enzyme-ligand equilibrium dissociation constant (K_D_)

The equilibrium dissociation constant for each tested compound from DDC was determined by monitoring the decrease in the 420 nm CD signal of DDC (7 µM) in the presence of various ligand concentrations in 100 mM potassium phosphate buffer, pH 7.4. In any case, the maximun DMSO concentration in the mixture was kept lower than 1% (v/v). The K_D_ values were obtained from the fitting of the data to the equation
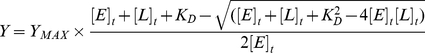
where [E]_t_ and [L]_t_ represent the total concentrations of DDC dimer and ligand, respectively, Y refers to the visible CD signal change at 420 nm at the ligand concentration [L], and Y_max_ refers to the CD signal when all enzyme molecules are complexed with the ligand. CD measurements were performed with a Jasco J-710 spectropolarimeter using quartz cuvettes with 1 cm path length.

## Supporting Information

Figure S1 **Normal distribution plot of predicted pK_i_ values for drug-like molecules.** 280000 drug-like molecules were docked in the active site of DDC with the Dovis 2.0 docking tool [23], [24] and the distribution of their predicted pK_i_ was derived. The normal distribution showed a mean pK_i_ of 5.57±0.81.
(TIF)

Figure S2 **Normal distribution plot of predicted pK_i_ values for lead-like molecules.** 180000 lead-like molecules were docked in the active site of DDC with the Dovis 2.0 docking tool [23], [24] and the distribution of their predicted pK_i_ was derived. The normal distribution showed a mean pKi of 5.30±0.62.
(TIF)

Figure S3 **Dose-response curve of the most potent compounds.** (A) Compounds obtained from the first and the second screening indicated with the following colors: black, magenta, light green, blue, orange, dark green and red, for compound 11, 17, 21, 23, 25, 34 and 37, respectively. (B) Compounds obtained from the third screening indicated with the following colors: dark green, purple, orange, magenta, blue, black, red and light green, for compound 39, 40, 41, 42, 43, 44, 45 and 46, respectively. Data were fitted using the standard IC_50_ equation with Origin 7.0 software (OriginLab).
(TIF)

Table S1 Ranking of the drug-like compounds, as assessed by VS protocol.
(DOC)

Table S2 Ranking of lead-like candidates, as assessed by VS protocol.
(DOC)

Table S3 Ranking of selected compounds, obtained by applying the similarity search of the entire ZINC database (∼9.0×10^6^ compounds), using compound **11** as query.
(DOC)

Table S4 Ranking of selected compounds, obtained by applying the similarity search of the entire ZINC database (∼9.0×10^6^ compounds), using compound **5** as query.
(DOC)
