# Supplementary figures and images for: Identification by Virtual Screening and In Vitro Testing of Human DOPA Decarboxylase Inhibitors
Source: PLoS One. 2012 Feb 23;7(2):e31610. doi: 10.1371/journal.pone.0031610 (PMC3285636; doi:10.1371/journal.pone.0031610)

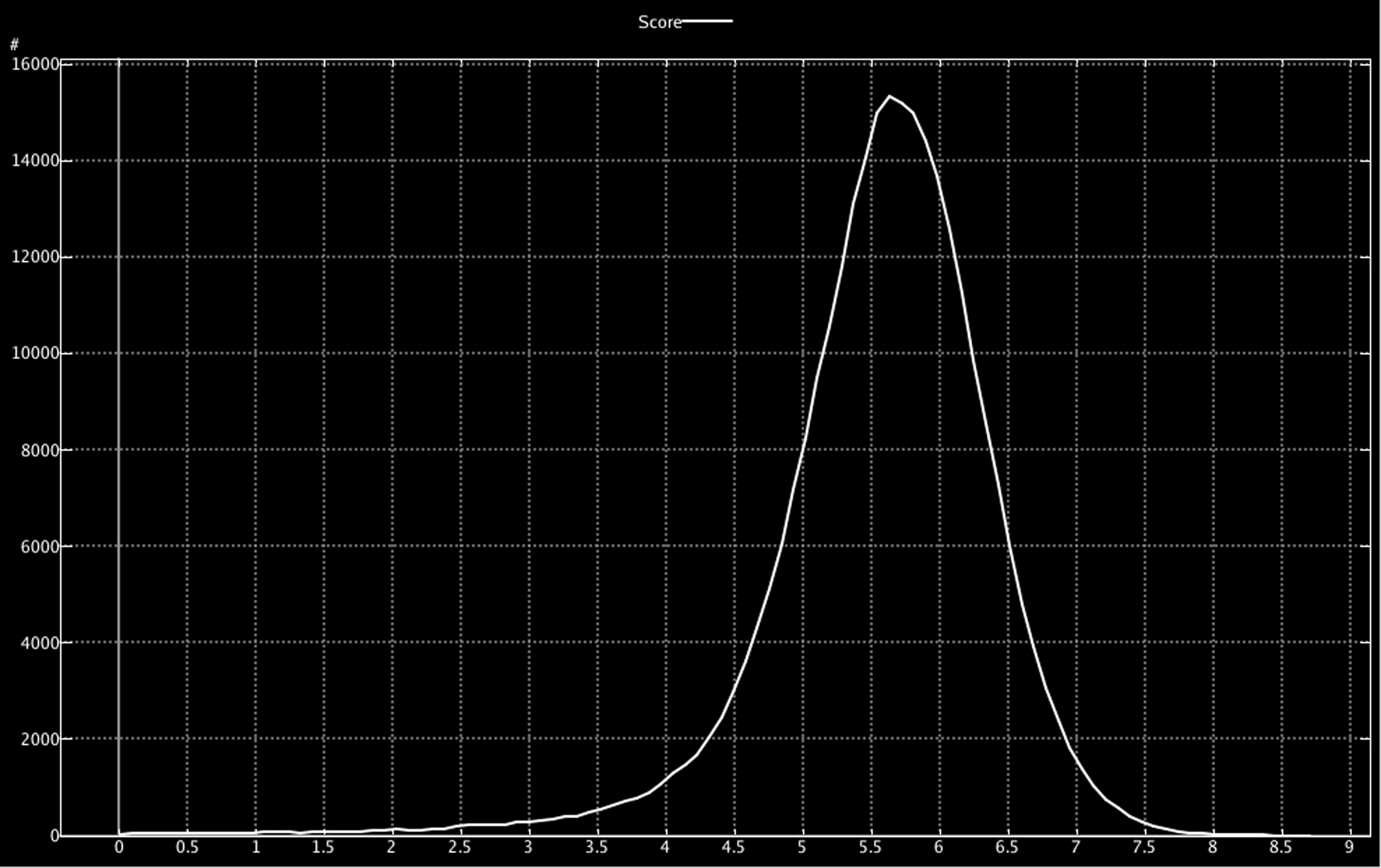

Supplement: Figure S1 — Normal distribution plot of predicted pKi values for drug-like molecules. 280000 drug-like molecules were docked in the active site of DDC with the Dovis 2.0 docking tool [23], [24] and the distribution of their predicted pKi was derived. The normal distribution showed a mean pKi of 5.57±0.81. (TIF) [file pone.0031610.s001.tif]

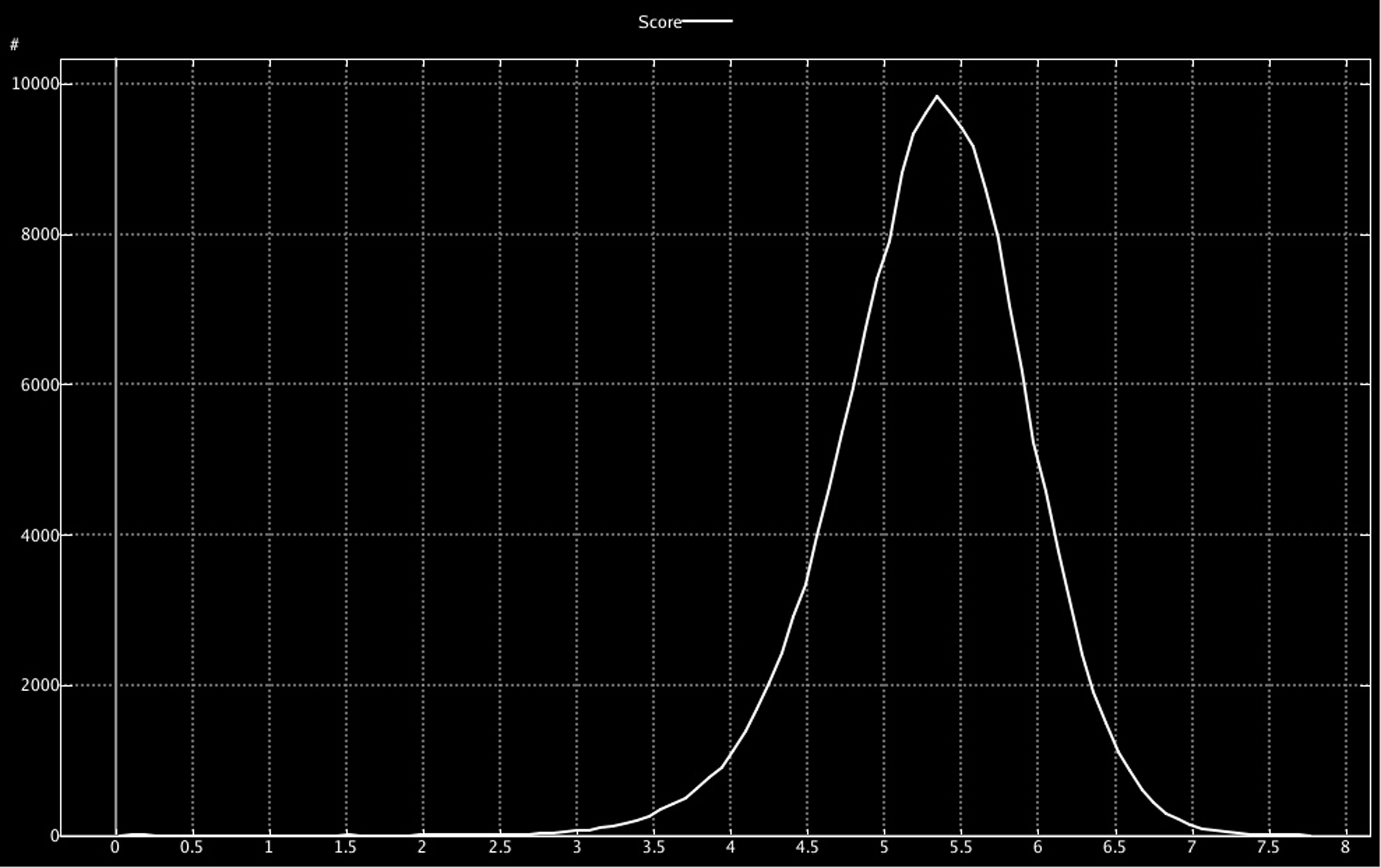

Supplement: Figure S2 — Normal distribution plot of predicted pKi values for lead-like molecules. 180000 lead-like molecules were docked in the active site of DDC with the Dovis 2.0 docking tool [23], [24] and the distribution of their predicted pKi was derived. The normal distribution showed a mean pKi of 5.30±0.62. (TIF) [file pone.0031610.s002.tif]

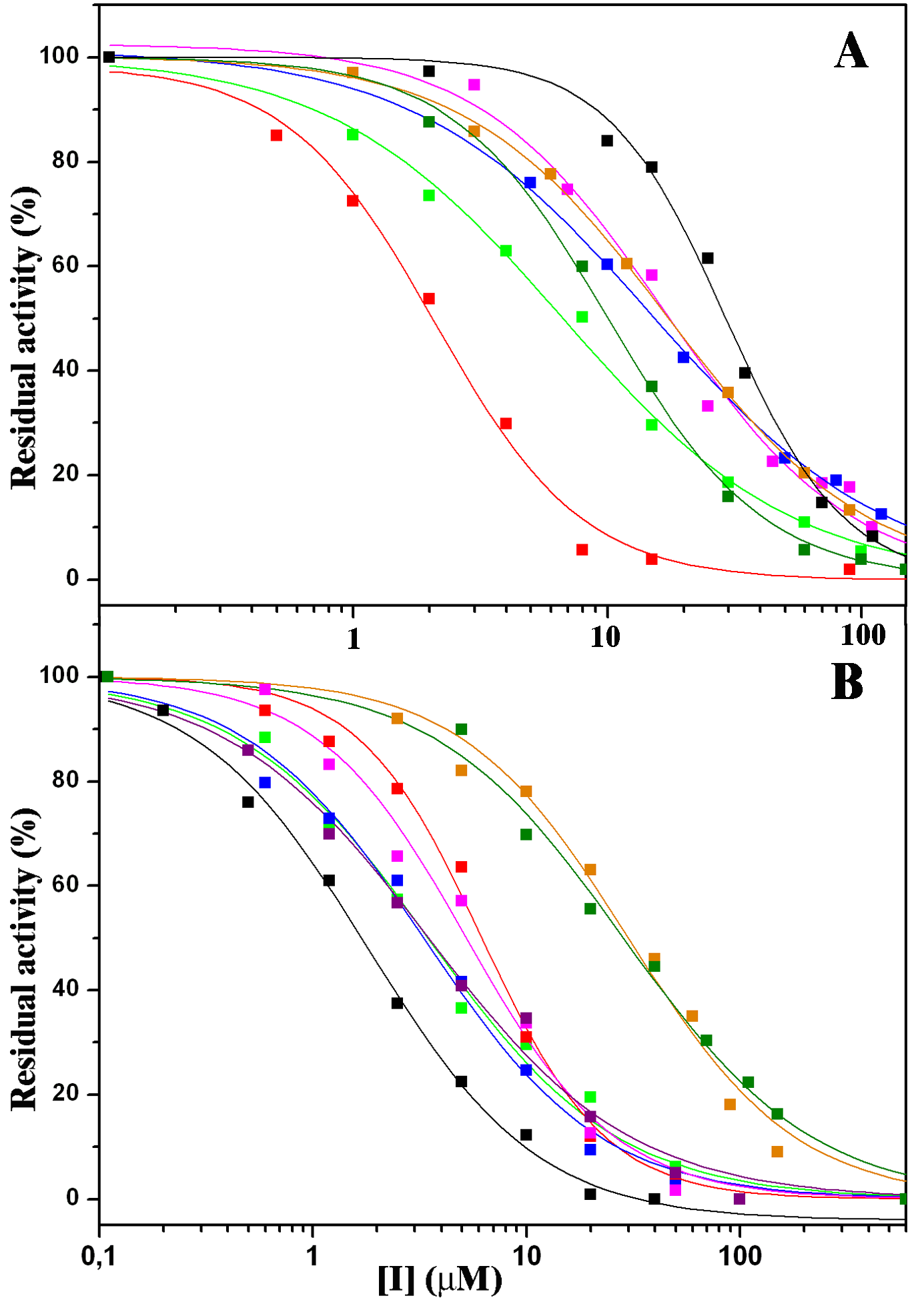

Supplement: Figure S3 — Dose-response curve of the most potent compounds. (A) Compounds obtained from the first and the second screening indicated with the following colors: black, magenta, light green, blue, orange, dark green and red, for compound 11, 17, 21, 23, 25, 34 and 37, respectively. (B) Compounds obtained from the third screening indicated with the following colors: dark green, purple, orange, magenta, blue, black, red and light green, for compound 39, 40, 41, 42, 43, 44, 45 and 46, respectively. Data were fitted using the standard IC50 equation with Origin 7.0 software (OriginLab). (TIF) [file pone.0031610.s003.tif]
