# Supplementary material for: Identification by Virtual Screening and In Vitro Testing of Human DOPA Decarboxylase Inhibitors
Source: PLoS One. 2012 Feb 23;7(2):e31610. doi: 10.1371/journal.pone.0031610 (PMC3285636; doi:10.1371/journal.pone.0031610)
Supplement: Table S1 — Ranking of the drug-like compounds, as assessed by VS protocol. (DOC) [file pone.0031610.s004.doc]

**Table S1.** Ranking of the drug-like compounds, as assessed by VS protocol.

| **Rank** | **ZINC code** | **Predicted** Mean Dissociation Constant **(nM)** |
| --- | --- | --- |
| 1 | ZINC11548105 | 23.85 |
| 2 | ZINC05936951* | 25.22 |
| 3 | ZINC08782681 | 38.39 |
| 4 | ZINC08161228 | 42.85 |
| 5 | ZINC01874906* | 43.66 |
| 6 | ZINC10319090 | 50.98 |
| 7 | ZINC02403139 | 53.17 |
| 8 | ZINC13036474 | 61.55 |
| 9 | ZINC01785030 | 62.47 |
| 10 | ZINC13036477 | 62.57 |
| 11 | ZINC13036468 | 63.91 |
| 12 | ZINC10215375 | 64.12 |
| 13 | ZINC08042801* | 65.66 |
| 14 | ZINC10441272 | 69.55 |
| 15 | ZINC13036479 | 70.11 |
| 16 | ZINC11335847 | 70.32 |
| 17 | ZINC13036470 | 77.74 |
| 18 | ZINC13036462 | 99.70 |
| 19 | ZINC07349578 | 101.10 |
| 20 | ZINC10899232 | 119.56 |
| 21 | ZINC00850864 | 120.48 |
| 22 | ZINC12526366 | 125.61 |
| 23 | ZINC09116258 | 127.33 |
| 24 | ZINC10927913 | 136.14 |
| 25 | ZINC12526266 | 142.46 |
| 26 | ZINC05658136 | 145.23 |
| 27 | ZINC05252711 | 148.57 |
| 28 | ZINC07709856 | 154.71 |
| 29 | ZINC11265957 | 156.56 |
| 30 | ZINC05252718* | 157.28 |
| 31 | ZINC12535964 | 162.01 |
| 32 | ZINC13036472 | 162.95 |
| 33 | ZINC13036474 | 175.94 |
| 34 | ZINC02897153 | 179.01 |
| 35 | ZINC12535964 | 180.42 |
| 36 | ZINC07726624 | 180.72 |
| 37 | ZINC07986167* | 194.35 |
| 38 | ZINC12526266 | 211.45 |
| 39 | ZINC12698878 | 240.47 |
| 40 | ZINC12603254* | 253.62 |
| 41 | ZINC07364465 | 303.64 |
| 42 | ZINC05185571* | 433.81 |
| 43 | ZINC12902180 | 525.29 |
| 44 | ZINC05298759 | 697.02 |

* purchased compounds
