# Supplementary material for: Identification by Virtual Screening and In Vitro Testing of Human DOPA Decarboxylase Inhibitors
Source: PLoS One. 2012 Feb 23;7(2):e31610. doi: 10.1371/journal.pone.0031610 (PMC3285636; doi:10.1371/journal.pone.0031610)
Supplement: Table S2 — Ranking of lead-like candidates, as assessed by VS protocol. (DOC) [file pone.0031610.s005.doc]

**Table S2.** Ranking of lead-like candidates, as assessed by VS protocol.

| **Rank** | **ZINC code** | **Predicted** Mean Dissociation Constant **(nM)** |
| --- | --- | --- |
| 1 | ZINC00342069* | 91.34 |
| 2 | ZINC01509851 | 114.84 |
| 3 | ZINC00492694* | 139.14 |
| 4 | ZINC01713053 | 148.60 |
| 5 | ZINC01736134 | 156.17 |
| 6 | ZINC00111962* | 158.53 |
| 7 | ZINC02654620 | 166.00 |
| 8 | ZINC03105437 | 226.60 |
| 9 | ZINC00518559 | 256.83 |
| 10 | ZINC00408890* | 266.62 |
| 11 | ZINC00242969 | 283.41 |
| 12 | ZINC02654612* | 373.37 |
| 13 | ZINC02653945 | 376.11 |
| 14 | ZINC03243721* | 486.91 |
| 15 | ZINC00134865* | 619.76 |
| 16 | ZINC03324540* | 642.08 |
| 17 | ZINC00317485 | 2501.52 |

*purchased compounds
