# Supplementary material for: Identification by Virtual Screening and In Vitro Testing of Human DOPA Decarboxylase Inhibitors
Source: PLoS One. 2012 Feb 23;7(2):e31610. doi: 10.1371/journal.pone.0031610 (PMC3285636; doi:10.1371/journal.pone.0031610)
Supplement: Table S3 — Ranking of selected compounds, obtained by applying the similarity search of the entire ZINC database (∼9.0×10^6^ compounds), using compound 11 as query. (DOC) [file pone.0031610.s006.doc]

**Table S3.** Ranking of selected compounds, obtained by applying the similarity search of the entire ZINC database (~ 9.0x10^6^ compounds), using compound **11** as query.

| **Rank** | **ZINC code** | **TC relative to compound 13** |
| --- | --- | --- |
| 6 | ZINC01478099 | 0.962264 |
| 8 | ZINC00550832 | 0.962264 |
| 9 | ZINC00520318 | 0.962264 |
| 34 | ZINC00530774 | 0.925926 |
| 38 | ZINC04898088 | 0.924528 |
| 40 | ZINC01183732 | 0.924528 |
| 43 | ZINC00553626 | 0.924528 |
| 62 | ZINC01473651 | 0.909091 |
| 63 | ZINC00548914 | 0.909091 |
| 72 | ZINC01184616 | 0.90566 |
| 74 | ZINC01183789 | 0.90566 |
| 76 | ZINC00554457 | 0.90566 |
| 78 | ZINC00549544 | 0.90566 |
| 127 | ZINC00532465 | 0.888889 |
| 134 | ZINC00344577 | 0.886792 |
| 276 | ZINC03044871 | 0.857143 |
| 320 | ZINC00199179 | 0.849057 |
| 440 | ZINC00344575 | 0.836364 |
| - | PB-05713794* | 0.923077 |
| - | STOCK3S-20068* | 0.924528 |

*Ambinter-code
