# Supplementary material for: Identification by Virtual Screening and In Vitro Testing of Human DOPA Decarboxylase Inhibitors
Source: PLoS One. 2012 Feb 23;7(2):e31610. doi: 10.1371/journal.pone.0031610 (PMC3285636; doi:10.1371/journal.pone.0031610)
Supplement: Table S4 — Ranking of selected compounds, obtained by applying the similarity search of the entire ZINC database (∼9.0×10^6^ compounds), using compound 5 as query. (DOC) [file pone.0031610.s007.doc]

**Table S4.** Ranking of selected compounds, obtained by applying the similarity search of the entire ZINC database (~ 9.0x10^6^ compounds), using compound **5** as query.

| **Rank** | **ZINC code** | **TC relative to compound 6** |
| --- | --- | --- |
| 26 | ZINC22621067 | 0.887324 |
| 656 | ZINC03886086 | 0.810811 |
